# Identification of potential *Campylobacter jejuni* genes involved in biofilm formation by EZ-Tn5 Transposome mutagenesis

**DOI:** 10.1186/s13104-017-2504-1

**Published:** 2017-05-12

**Authors:** Amy Huei Teen Teh, Sui Mae Lee, Gary A. Dykes

**Affiliations:** 1 School of Science, Monash University, Jalan Lagoon Selatan, Bandar Sunway, 46150 Selangor Darul Ehsan Malaysia; 2 School of Public Health, Curtin University, Bentley, WA 6102 Australia

**Keywords:** Biofilm, *Campylobacter jejuni*, Transposon mutagenesis, EZ-Tn5 Transposome

## Abstract

**Background:**

Biofilm formation has been suggested to play a role in the survival of *Campylobacter jejuni* in the environment and contribute to the high incidence of human campylobacteriosis. Molecular studies of biofilm formation by *Campylobacter* are sparse.

**Results:**

We attempted to identify genes that may be involved in biofilm formation in seven *C. jejuni* strains through construction of mutants using the EZ-Tn5 Transposome system. Only 14 mutants with reduced biofilm formation were obtained, all from one strain of *C. jejuni*. Three different genes of interest, namely CmeB (synthesis of multidrug efflux system transporter proteins), NusG (transcription termination and anti-termination protein) and a putative transmembrane protein (involved in membrane protein function) were identified. The efficiency of the EZ::TN5 transposon mutagenesis approach was strain dependent and was unable to generate any mutants from most of the strains used.

**Conclusions:**

A diverse range of genes may be involved in biofilm formation by *C. jejuni*. The application of the EZ::TN5 system for construction of mutants in different *Campylobacter* strains is limited.

**Electronic supplementary material:**

The online version of this article (doi:10.1186/s13104-017-2504-1) contains supplementary material, which is available to authorized users.

## Background

*Campylobacter jejuni* is a Gram negative, microaerophilic bacterial species with fastidious temperature, atmosphere and nutrient growth requirements [1]. Despite these requirements, *C. jejuni* is one of the most frequent causes of human bacterial gastrointestinal food-borne infection worldwide [2]. Common symptoms associated with infection by this pathogen include diarrhea, fever and abdominal pain which may also lead to more serious neuropathies such as Guillain–Barre and Miller–Fisher syndromes [3, 4].

*Campylobacter jejuni* is widely spread in the environment and can be readily isolated from food, water and other sources [5, 6]. Biofilm formation has been suggested to help *C. jejuni* overcome the disadvantages of survival in the environment by protecting the bacteria from various stressors [7, 8].

While biofilm formation by other bacterial species, such as *Pseudomonas aeruginosa* and *Escherichia coli* are well studied, molecular studies of biofilm formation by *Campylobacter* are sparse. A study carried out by Reeser et al. [9] showed that flagella plays a role in biofilm formation by *Campylobacter*. These authors reported that flagellum-deficient mutant strains (*flaAB* mutant strains) showed lower levels of biofilm formation. In addition, biofilm formation by *Campylobacter* was impaired in strains defective in a putative flagellar protein (FliS) and in phosphate acetyltransferase (Cj0688) [10]. A study carried out by Fields, Thompson [11] showed that biofilm formation was lowered in *C. jejuni* strains with a mutated CsrA (carbon starvation regulator) gene. The product of this gene in other bacterial species has been identified as a posttranscriptional regulator of translation responsible for repression or activation of many important processes, including regulation of the stress response [11, 12]. A study by Oh and Jeon [13] showed that biofilm formation by *C. jejuni* was increased in strains with a mutated alkyl hydroperoxide reductase (AhpC) gene, which is involved in the oxidative stress response.

Although there is evidence for the role of flagella and gene regulation in biofilm formation, molecular understanding of biofilm formation by *C. jejuni* is still in its infancy. In this study, we attempted to identify novel genes that may be involved in biofilm formation by *C. jejuni* through random mutagenesis as this is established as a useful tool in deducing gene function [14]. Since transposons have been widely used in the construction of mutant libraries due to their ability to randomly insert into the genome, an in vivo transposition system, the EZ-Tn5 Transposome (Epicentre, USA), was used in this study. The EZ::TN5 transposome can be generated in vitro using purified EZ::TN5 transposase and a DNA fragment (usually antibiotic cassette) flanked by inverted repeats. This system has been shown to be an efficient and reliable method of random insertion of transposon DNA into the genome of many different microorganisms in numerous studies [14–16].

## Methods

### Bacterial strains and growth conditions

Seven *C. jejuni* strains (2862, 2863, 2865, 2866, 2868, 2869 and 2871) isolated from poultry obtained from retail outlets in Malaysia as reported by Wieczorek et al. [17] were used in this study. *Campylobacter jejuni* ATCC 33291 obtained from the American Type Culture Collection was also used in this study. Whole genomes of three of the strains, 2865, 2868 and 2871, were sequenced as described in Teh et al. [18]. All the strains were maintained at −80 °C in Nutrient Broth No. 2 (NB2, Oxoid, UK) and 15% glycerol and were resuscitated on *Campylobacter* blood-free selective agar base (Oxoid, UK) (as sessile cultures) with incubation at 37 °C for 48 h under microaerobic conditions generated using Campygen (Oxoid, UK).

### Construction of transposon mutants of *C. jejuni*

#### EZ-Tn5 transposome preparation

An attempt was made to construct a transposon library for each of eight *C. jejuni* strains using the EZ-Tn5 transposase and the EZ-Tn5 pMOD-3<R6Kγori/MCS> transposon construction vector (Epicenter Biotechnologies, USA) in vivo according to the manufacturer’s instructions. A chloramphenicol (Cm) resistance cassette was amplified from pBACe3.6 (GenBank Accession No. U80929) [19] using primers CmF (5′-GAATTCGATCGGCACGTAAGAGGTTC-3′) and CmR (5′-AAGCTTGGGCACCAATAA CTGCCTTA-3′) which resulted in a PCR product of 788 bp. The purified Cm resistance gene cassette was then ligated into pGEM-T vector (Promega, USA) and transformed into Top-10 *E. coli* competent cells and plated on Luria–Bertani (LB) plate supplemented with 30 µg/mL Cm. The pGEM-T::Cm was extracted from positive clones using the PureLink Quick Plasmid Miniprep Kit (Invitrogen, USA) and subjected to restriction digestion with *Eco*RI and *Hin*dIII before cloning into *Eco*RI and *Hin*dIII-digested EZ-Tn5 pMOD-3<R6 Kγori/MCS> to yield the EZ-Tn5-Cm transposon vector. The vector was then transformed into *E. coli* Top-10 competent cells and plated on LB plate supplemented with 30 µg/mL Cm. Positive clones were selected and plasmids were extracted. The EZ-Tn5-Cm transposon region was amplified by PCR using primers PCRFP (5′-ATTCAGGCTGCGCAACTGT-3′) and PCRRP (5′-GTCAGTGAGCGAGGAAGCGGAAG-3′) and purified. EZ-Tn5 Transposomes was prepared by adding 2 µL of EZ-Tn5 transposon DNA, 4 µL of EZ-Tn5 transposase and 2 µL of glycerol. The reaction mixture was incubated for 30 min at room temperature. The resulting mixture was stored at −20 °C and used for mutagenesis of *C. jejuni.*

#### Preparation of electrocompetent cells

Electrocompetent *C. jejuni* strains were prepared as described previously [20]. Briefly, bacteria from a lawn grown overnight on Skirrow agar were harvested into 2 mL Mueller–Hinton (MH) broth, pelleted at 3220×*g* for 20 min at 4 °C, and resuspended in 2 mL ice-cold wash buffer (272 mM sucrose, 15% glycerol). The washing step was repeated for three times and the bacteria were then resuspended in 1 mL ice-cold wash buffer. Cells were aliquoted in 100 μL and stored at −80 °C until needed.

#### Electroporation conditions

Electroporation was performed as described previously [20]. One milliliter of the transposome was added into 100 μL *C. jejuni* electrocompetent cells in a 0.2 cm electroporation cuvette on ice and gently mixed. Electroporation was performed with a Bio-Rad MicroPulser (2.5 kV, 600 Ω, and 10 μF) (Bio-Rad, USA). Following electroporation, 200 μL of SOC broth (2% Bacto Tryptone (Difco, US), 0.5% yeast extract (Difco, US), 10 mM NaCl, 2.5 mM KCl, 10 mM MgCl_2_, 10 mM MgSO_4_, 20 mM glucose) was added to the electroporated cells and the mixture spread onto *Campylobacter* blood-free selective agar plates. Plates were incubated for 24 h microaerobically, and bacteria were then harvested into 1 mL NB2 broth, centrifuged at 10,000×*g* for 2 min, resuspended in 100 μL NB2 broth, and plated onto *Campylobacter* blood-free selective agar containing 30 µg/mL Cm to select for transformants. Colonies were individually patched to fresh *Campylobacter* blood-free selective agar-Cm plates to confirm resistance, followed by inoculation into NB2 broth, growth for 2 days under microaerobic conditions, and storage at −80 °C in 15% glycerol.

### Assessment of biofilm formation

The ability of the original *C. jejuni* strains and transposon mutants to form biofilm was determined in 96-well polystyrene microtiter plates (TPP^®^, Switzerland) using Mueller–Hinton broth (MHB) and Brucella broth under microaerobic and aerobic conditions by the methods described by Skyberg et al. [21] with slight modifications. Briefly, the strains were grown as sessile cultures under microaerobic conditions for 48 h at 37 °C. After incubation, the colonies on the agar plates were harvested by suspending in 5 mL of phosphate buffer saline (PBS; 1st BASE, Singapore). The cells were then diluted 1:10 in MHB or Brucella broth (~10^7^ CFU/mL) and a 200 µL aliquot of each dilution was transferred to a microtiter plate well. Six wells per microtiter plate were used for each strain and a further six wells were filled with uninoculated medium which serve as negative control. The plates were then incubated at 37 °C for 6 days under microaerobic or aerobic conditions without shaking. The assays were performed in triplicate. The wells were then examined using the crystal violet and the absorbance was determined using a microplate reader (Tecan, Switzerland) at 550 nm.

### Determination of EZ-Tn5-Cm insertion sites

Genomic DNA was extracted from the mutants impaired in biofilm formation using Wizard Genomic DNA Purification Kit (Promega, USA). This DNA was used as the template for a single-primer PCR amplification known as RATE (Random Amplification of Transposon Ends) as described previously [15]. PCR amplification was carried out using SqFP (5′-GCCAACGACTACGCACTAGCC AAC-3′), which binds within the transposon. The PCR conditions used were as follow: 30 cycles at 95 °C for 30 s, 55 °C for 30 s, and 72 °C for 3 min; 30 cycles at 95 °C for 30 s, 30 °C for 30 s, and 72 °C for 2 min; and 30 cycles at 95 °C for 30 s, 55 °C for 30 s, and 72 °C for 2 min. The amplified PCR product was then sent for sequencing and the transposon insertion site was identified by Basic Local Alignment Search Tool (BLAST) analysis of the DNA sequence immediately flanking the mosaic end of the transposon [22].

### Statistical analysis

Biofilm formation assays were performed in triplicate using independently grown cultures. All the statistical analysis was performed using SPSS 18 software. Data obtained was analyzed using one way analysis of variance (ANOVA) and pairwise comparisons of the means were conducted using Tukey’s post hoc test at a 95% confidence level.

## Results and discussion

### Transposon mutagenesis of *C. jejuni*

In order to identify the genes that are involved in biofilm formation, an attempt was made to construct a transposon library for each of eight *C. jejuni* strains by transposon mutagenesis and the ability of individual mutants to form biofilm was then screened and compared to that of the original strain. A stable transposome complex consisting of EZ-Tn5 transposons containing a chloramphenicol resistance cassette (EZ-Tn5 <Cm> transposon) was constructed in vitro in the absence of Mg^2+^. The transposomes formed were then electroporated into *C. jejuni* competent cells where the transposase was activated by intracellular Mg^2+^ leading to random insertion of the transposon into *C. jejuni* genomic DNA. This in vivo protocol successfully generated Cm^R^ mutants in *C. jejuni* 2868. The number of mutants generated was, however, very low with only 37 mutants recovered. Out of these 37 mutants, only 22 remained viable when resuscitated from frozen stock. No mutant was generated from the other seven strains used in this study. These results suggest that the efficiency of transposon mutagenesis is strain-dependent. A similar finding was reported in a previous study where the EZ-Tn*5* transposon system successfully generated a large number of mutants in *C. jejuni* 81–176 but a low number or no mutants were obtained from other *C. jejuni* strains including the *C. jejuni* ATCC 33291 strain used in our study [16]. This result may be due to the presence of specific restriction–modification systems in selected *C. jejuni* strains, especially the wild type strains used in this study. Previous studies have shown that the EZ-Tn5 transposon system successfully generated transposition mutants effectively in different bacterial species including *Escherichia coli* [23]*, Salmonella enterica* serovar Typhi [24]*, Neisseria gonorrhoeae* [15] and *Bacteroides fragilis* [25]. The results obtained from this study, however, indicate that the potential for application of in vivo transposon mutagenesis in *Campylobacter* strains might be limited due to this strain-dependent transposition efficiency [16].

### Screening of biofilm forming ability of the mutant strains

**Fig. 1:**
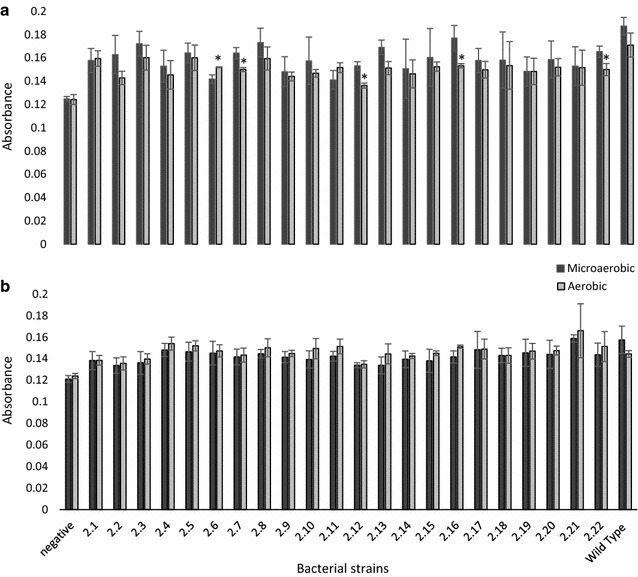
Comparison of biofilm formation by *Campylobacter jejuni* 2868 mutant strains, strains 2.1–2.22 obtained through transposon mutagenesis using the EZ-Tn5 Transposome mutagenesis system, grown in **a** Mueller–Hinton Broth; **b** Brucella Broth and incubated for 6 days at 37 °C. The negative is uninoculated medium. All results are presented in mean ± SD where n = 3; Symbol *asterisk* indicates significant difference on biofilm formed under microaerobic and aerobic conditions within the same bacterial strain where p < 0.05

All the 22 mutant strains generated from strain 2868 were screened for biofilm formation and the results are shown in Fig. 1. In general, levels of biofilm formed in MH broth were significantly higher (p < 0.05) as compared to Brucella broth as shown in the results of one way ANOVA with Tukey’s post hoc test presented in Additional file 1: Table S1. In addition, most of the mutant strains showed no significant difference (p > 0.05) in biofilm formation when incubated in MH under different oxygen conditions. Nevertheless, 4 out of 22 strains showed a significant decrease (p < 0.05) while one strain showed a significant increase (p < 0.05) in biofilm formation under aerobic condition as compared to microaerobic condition. This suggested that the gene that was disrupted in these mutant strains may be involved in the oxidative stress response. On the other hand, all the strains showed no significant difference (p > 0.05) in biofilm formation when incubated under aerobic or microaerobic conditions in Brucella broth. This may be due to the presence of sodium bisulfite in Brucella broth which acts as a reducing agent and protects the bacteria from oxygen stress when incubated under aerobic condition. This is consistent with previous study which showed that the presence of supplement containing reducing agents, for example sodium metabisulphite and sodium pyruvate, minimized the conversion of rods to coccoid forms of *C. jejuni,* which occur when conditions are unfavourable for its growth [26].

### Determination of genes disrupted in the mutants with decreased biofilm formation

Since most of the strains showed higher biofilm formation in MH under microaerobic conditions, a one way ANOVA was carried out to establish if the biofilm forming ability of the mutant strains was different as compared to the wild type strain under these conditions. The results showed that 14 out of the 22 mutant strains (strains 2.1, 2.4–2.7, 2.9, 2.11–2.13, 2.17, 2.19–2.22) showed significantly lower (p < 0.05) biofilm formation as compared to the wild type strain. The reduction in biofilm formation was, however, low as compared to other studies that investigated the role of specific genes on biofilm formation by *C. jejuni* through construction of different mutant strains [10, 11]. This might be due to the originally low biofilm formation in the wild type strain (strain 2868) used in our study, which may be a common occurrence among *C. jejuni* in general [27].

The insertion site of EZ-Tn5 <Cm> transposon in the 14 mutants above were identified through RATE (Random Amplification of Transposon Ends) PCR and the sequences obtained were subjected to BLAST and were compared to the available *C. jejuni* NCTC 11168 genomic sequence [28] to identify the mutated gene. The location of transposons in 4 out of the 14 mutants occurred in coding regions and the genes disrupted are listed in Table 1. It is likely that the transposons inserted in non-coding regions in the other mutants as no genes could be identified.

**Table 1:** Identification of EZ-Tn5 <Cm> transposon insertion sites in *C. jejuni* 2868 mutants with decreased biofilm formation

Mutant strain	Locus designation	Gene	Putative gene function
2.9	N/A	CmeB	Transporter protein, part of a multidrug efflux system
2.11	Cj0473	NusG	NusG is a bacterial transcriptional elongation factor involved in transcription termination and anti-termination
2.19	Cj0268c	Membrane protein/transmembrane protein	Probable transmembrane protein

N/A not available

Genes disrupted in mutants displaying decreased biofilm formation were identified as CmeB (multidrug efflux system transporter protein synthesis), NusG (transcription termination and anti-termination) and a putative transmembrane protein (involved in membrane protein function). Several studies have shown that defective in efflux pump activity impairs biofilm formation in different bacterial species (*Escherichia coli*, *Klebsiella, Salmonella*, *Pseudomonas aeruginosa* and *Staphylococcus aureus*) [29–32]. This may also contribute to lower biofilm formation in mutant strain 2.9 in this study. The presence of hydrophobic components on the surface of bacterial cells will contribute to cell surface hydrophobicity and promote bacteria adhesion and contribute to biofilm formation [33, 34]. Similarly, capsular polysaccharides (CPS) and lipooligosachharides (LOS) will affect cell surface hydrophobicity, auto-aggregation and attachment of many *Campylobacter* strains to cell lines and abiotic surfaces [35–37]. This may indicate that the gene involved in membrane protein synthesis may affect the cell integrity of the outer membrane of mutant strain 2.19 which in turn may affect its biofilm formation.

Overall, our study indicated that the utilization of an in vivo transposon mutagenesis approach using the EZ::TN5 system could, in principle, identify genes involved in biofilm formation by *C. jejuni*. The role of these genes in biofilm formation is, at this stage, only putative, and further studies, including gene complementation, need to be carried out to confirm their role. The strains and growth conditions used in this study for biofilm formation were established as suitable in previous studies [38, 39], however, a combination of different strains and growth conditions may result in higher biofilm formation and potentially different outcomes.

Notably, our study also showed that the EZ::TN5 system was unable to generate mutants from most of the strains used. Furthermore, the relative inefficiency in generating mutants in strains in which this can occur means that repeating the process until sufficient mutants are gathered may be required. This suggested that the efficiency of this transposon mutagenesis approach was strain dependent and its application is likely limited for use in *C. jejuni.*

## Additional file

**Additional file 1: Tables S1.** One way ANOVA and Tukey’s Post Hoc comparison showing the biofilm formation by the *Campylobacter jejuni* strains in different type of media (Muller-Hinton or Brucella broth) incubated under different oxygen conditions (aerobic or microaerobic).

## Abbreviations

ANOVA: analysis of variance
ATCC: American type culture collection
BLAST: Basic local alignment search tool
CFU: colony forming unit
Cm: chloramphenicol
MH: Mueller–Hinton
NB2: Nutrient Broth No. 2
PBS: phosphate buffer saline
PCR: polymerase chain reaction
RATE PCR: random amplification of transposon ends polymerase chain reaction
SD: standard deviation
