# Supplementary material for: Identification of potential Campylobacter jejuni genes involved in biofilm formation by EZ-Tn5 Transposome mutagenesis
Source: BMC Res Notes. 2017 May 12;10:182. doi: 10.1186/s13104-017-2504-1 (PMC5427567; doi:10.1186/s13104-017-2504-1)
Supplement: Supplementary file 1 — Additional file 1: Tables S1. One way ANOVA and Tukey’s Post Hoc comparison showing the biofilm formation by the Campylobacter jejuni strains in different type of media (Muller-Hinton or Brucella broth) incubated under different oxygen conditions (aerobic or microaerobic). [file 13104_2017_2504_MOESM1_ESM.docx]

**Additional file: Table S1:** One way ANOVA and Tukey’s Post Hoc comparison showing the biofilm formation by the *Campylobacter jejuni* strains in different type of media (Muller-Hinton or Brucella broth) incubated under different oxygen conditions (aerobic or microaerobic).

|  | Sum of Squares | df | Mean Square | F | Sig. |
| --- | --- | --- | --- | --- | --- |
| Between Groups | .010 | 3 | .003 | 22.226 | .000 |
| Within Groups | .045 | 284 | .000 |  |  |
| Total | .055 | 287 |  |  |  |

| **Absorbance** | | | | |
| --- | --- | --- | --- | --- |
| Tukey HSD^a^ | | | | |
| Type of Media | N | Subset for alpha = 0.05 | | |
|  |  | 1 | 2 | 3 |
| Brucella (Microaerobic) | 72 | .141893 |  |  |
| Brucella (Aerobic) | 72 | .145652 | .145652 |  |
| MH (Aerobic) | 72 |  | .150540 |  |
| MH (Microaerobic) | 72 |  |  | .158020 |
| Sig. |  | .276 | .091 | 1.000 |
| Means for groups in homogeneous subsets are displayed. | | | | |
| a. Uses Harmonic Mean Sample Size = 72.000. | | | | |
